# Neuregulin-1 and ALS19 (ERBB4): at the crossroads of amyotrophic lateral sclerosis and cancer

**DOI:** 10.1186/s12916-024-03293-3

**Published:** 2024-02-19

**Authors:** Jacob J. Adashek, Chinmayi Pandya, Nicholas J. Maragakis, Pradip De, Philip R. Cohen, Shumei Kato, Razelle Kurzrock

**Affiliations:** 1 Department of Oncology, The Johns Hopkins Hospital, The Sidney Kimmel Comprehensive Cancer Center, Baltimore, MD USA; 2 Center for Personalized Cancer Therapy and Division of Hematology and Oncology, Department of Medicine, UC San Diego Moores Cancer Center, La Jolla, CA USA; 3 Department of Neurology, The Johns Hopkins Hospital, Baltimore, MD USA; 4 Cancer Genomics, Avera Cancer Institute, Sioux Falls, SD USA; 5 Department of Dermatology, Davis Medical Center, University of California, Sacramento, CA USA; 6 Touro University California College of Osteopathic Medicine, Vallejo, CA USA; 7 WIN Consortium, Paris, France; 8 MCW Cancer Center, Milwaukee, WI USA; 9 University of Nebraska, Omaha, NE USA

**Keywords:** ALS19, Amyotrophic lateral sclerosis, Cancer, ERBB4, Novel targets, NRG1, Targeted therapy

## Abstract

**Background:**

Neuregulin-1 (NRG1) is implicated in both cancer and neurologic diseases such as amyotrophic lateral sclerosis (ALS); however, to date, there has been little cross-field discussion between neurology and oncology in regard to these genes and their functions.

**Main body:**

Approximately 0.15–0.5% of cancers harbor *NRG1* fusions that upregulate NRG1 activity and hence that of the cognate ERBB3/ERBB4 (HER3/HER4) receptors; abrogating this activity with small molecule inhibitors/antibodies shows preliminary tissue-agnostic anti-cancer activity. Notably, ERBB/HER pharmacologic suppression is devoid of neurologic toxicity. Even so, in ALS, attenuated ERBB4/HER4 receptor activity (due to loss-of-function germline mutations or other mechanisms in sporadic disease) is implicated; indeed, ERBB4/HER4 is designated ALS19. Further, secreted-type NRG1 isoforms may be upregulated (perhaps via a feedback loop) and could contribute to ALS pathogenesis through aberrant glial cell stimulation via enhanced activity of other (e.g., ERBB1-3/HER1-3) receptors and downstream pathways. Hence, pan-ERBB inhibitors, already in use for cancer, may be agents worthy of testing in ALS.

**Conclusion:**

Common signaling cascades between cancer and ALS may represent novel therapeutic targets for both diseases.

## Background

Neuregulins, a family of epidermal growth factor (EGF)-like signaling molecules, are involved in cell-to-cell crosstalk and also participate in the development and repair of diverse body elements including those of the nervous system, skeletal muscle, heart, breast, and other organs [1–3]. The neuregulin family includes NRG1 (types I–VI), NRG2, NRG3, and NRG4.

The *NRG1* gene is located on the 8p12 region of the short arm of chromosome 8; it can translate to six different NRG1 (neuregulin1) protein types and over 30 different isoforms that act as extracellular EGF-like ligands for ERBB3/HER3 and ERRB4/HER41; the many different isoforms may be the reason why NRG1 can influence diverse functions such as proliferation and differentiation of glial, neuronal, and Schwann cells, expression of acetylcholine receptors in synaptic vesicles during neuromuscular junction formation, growth of skeletal muscle cells, lobuloalveolar budding/milk production in the breast, differentiation of breast cancer cells, and the development of the myocardium [4]. Neurohypophyseal NRG1 is derived from the hypothalamus as a prolactin modulator and is mainly expressed in rat pituitary gonadotrope cells and possibly regulates prolactin secretion in a juxtacrine manner [5, 6].

As mentioned, NRG1 binds to ERBB3/HER3 and ERBB4/HER4. ERBB3/HER3 lacks or has little intrinsic tyrosine kinase enzymatic activity; however, it frequently forms heterodimers with other ERBB/HER tyrosine kinases and, in cancer cells, can activate oncogenic signaling. While EGFR/ERBB1/HER1, ERBB3/HER3 and ERBB4/HER4 have ligands, ERBB2/HER2 has no known ligand. When a ligand binds to the extracellular region of EGFR/ERBB1/HER1, ERBB3/HER3, or ERBB4/HER4, the dimerization arm in domain II is exposed leading to receptor-receptor interaction; dimerization is a crucial step for receptor function and activation of cytoplasmic signaling [7]. In contrast, ERBB2/HER2 is always in a constitutively active conformation with its dimerization arm opening even without ligand binding.

**Fig. 1:**
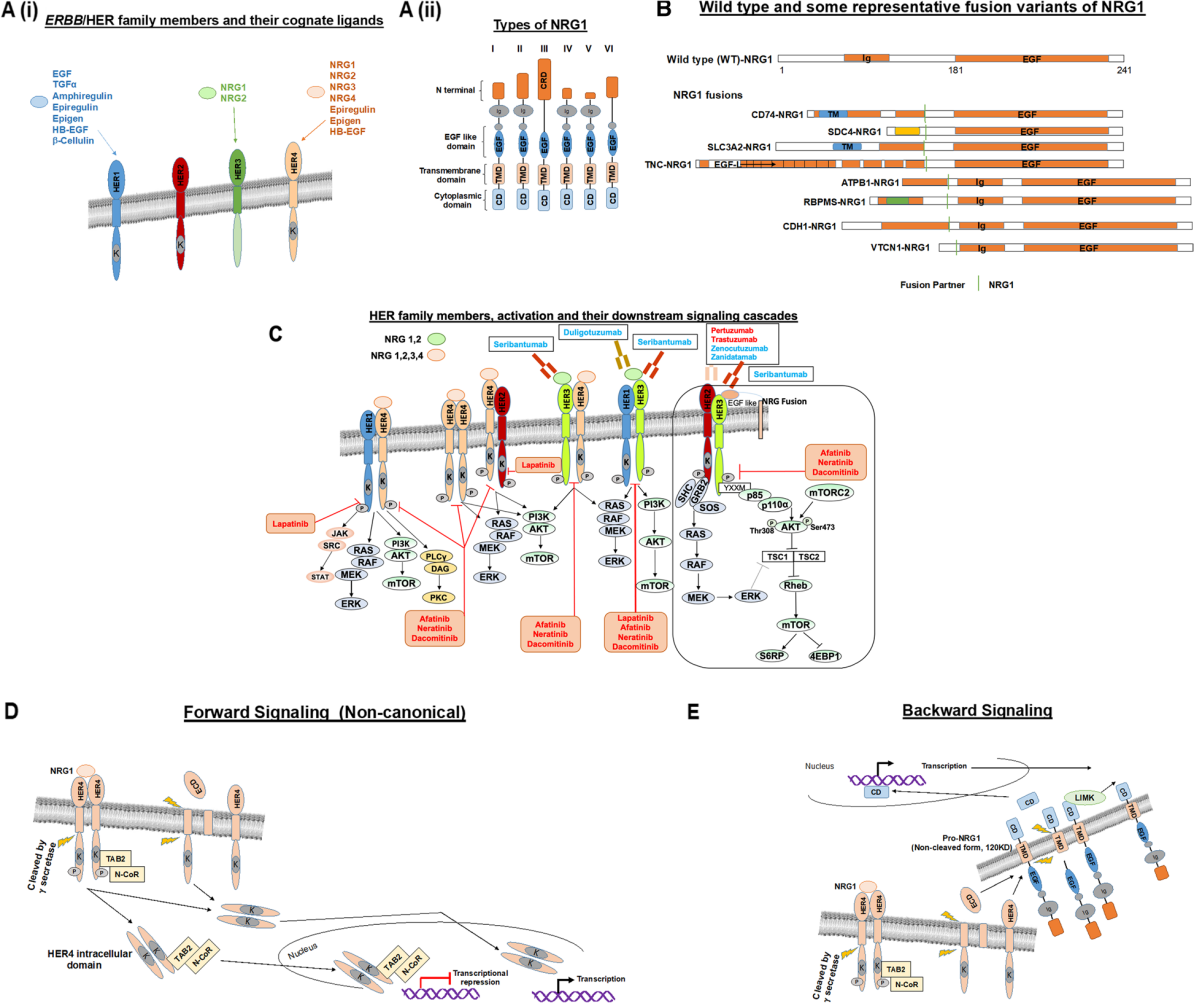
**A** (i) ERBB/HER family members and their cognate ligands (ii) Structural difference of various (I-VI) types of NRG1. Abbreviations: CD, cytoplastmic domain; CRD, Cysteine-rich domain; ECD, extracellular domain; EGF, epidermal growth factor; EGF-L; EGF-like repeat; HB-EGF, heparin-binding EGF-like growth factor; Ig, Ig-like C2-type domain; LIMK, LIM kinase; N-CoR, Nuclear receptor co-repressor; TA B2, TGF-Beta Activated Kinase 1 (MAP3K7) Binding Protein 2); TGF, transforming growth factor; TM, transmembrane; TMD, transmembrane domain; WT, wild type. **B** Examples of various fusions of *NRG1.* The structure of some representative variants of NRG1 fusions is shown. The EGF domain is preserved in all fusion proteins. **C** ERBB/HER family and potential downstream cascades. Figure represents possible sets for ERBB3/HER3 or ERBB4/HER4 dimerization with other ERBB/HER family members (HER1:HER4, HER1:HER3, HER2:HER4, HER4:HER4, HER3:HER4, and HER2:HER3) and their ligand(s) (e.g., NRG1, 2, 3, and 4) binding or their binding with EGF-like structure of NRG1 fusion-protein. Note that ERBB4/HER4 is also known as ALS19. NRG1 fusion-protein exerts a tumorigenic effect that requires HER2:HER3 heterodimerization-mediated activation, which can result in oncogenic signaling. The NRG1 fusion product is a transmembrane protein with an extracellular EGF-like domain that binds to ERBB3/HER3 in the cell membrane (see inside the box). NRG or NRG1-fusion-induced HER2:HER3 heterodimerization is depicted as the inset. The binding of ligands to receptors triggers dimerization and activation of the downstream signaling events responsible for tumorigenesis. Out of four family members, ERBB3/HER3 has six YXXM motifs responsible for the recruitment of p85, leading to activation of the PI3K-AKT-mTOR pathway. Other receptor dimerization also activates the RAS-RAF-MAPK pathway responsible for proliferation and survival. The NRG-HER signaling pathway also activates downstream JAK-STAT and PLCγ-PKC pathways, and both play a role in various oncogenic phenotypes. Examples of FDA-approved drugs are shown in the red color font, and examples of non-approved drugs are presented in blue font (inside the box). ERBB2/HER2 may also interact with ERBB3/HER3 and IGF1R to form heterotrimeric complex (HER2-HER3-IGF1R) [not shown in the figure] in trastuzumab-resistant breast cancer cells. **D** NRG1-mediated ERBB4/HER4 activation forward signaling (non-canonical). Non-canonical ERBB4/HER4 (also known as ALS19) forward signaling is shown. The ERBB4/HER4 intracellular domain is cleaved by γ-secretase (or others) (separated from the extracellular domain (ECD); the ERBB4/HER4 intracellular domain translocates to the nucleus to regulate gene expression. Also, NRG1-mediated HER4 activation (phosphorylation) promotes the association with an adaptor protein TA B2. TA B2 also recruits N-CoR and forms a signaling complex that, upon translocation to the nucleus, represses the transcription of certain genes required for the differentiation of neuronal precursor cells. **E** NRG1- mediated backward signaling. For NRG1-mediated backward signaling, the C-terminal fragment of NRG1 (CD: cytoplasmic domain) is cleaved from the Pro-NRG1 by the help of a protease; NRG1 CD may translocate to the nucleus to regulate gene transcription. The CD of Pro-NRG1 also interacts with LIM kinase (LIMK). LIMK (a non-receptor tyrosine kinase) has been shown to regulate cytoskeletal rearrangement/actin dynamics in many cell types including neuronal cells. In addition, ERBB4/HER4 (ALS19) or its diffusible extracellular domain can act as a ligand for pro-NRG1

Importantly, the NRG family, of which NRG1 is a member, includes three other subtypes (NRG-2 (Don-1, NTAK), NRG-3, and NRG-4), each with unique functionality profiles and binding sites (Fig. 1 panels A–F; Table 1) [1, 4, 8–20]. Specifically, NRG1-2 serve as ligands that bind to ERBB3/HER3, and NRG1-4 bind to ERBB4/HER4 (Fig. 1A). Because ERBB3/HER3 and ERBB4/HER4 can each heterodimerize with EGFR/ERBB1/HER1 and ERBB2/HER2 (and with each other), NRG1 can also indirectly affect the function of EGFR/ERBB1/HER1 and ERBB2/HER2 through ERBB3/HER3 and ERBB4/HER4 by recruiting the former co-receptors, resulting in ligand-induced tyrosine phosphorylation. Depending on which receptor NRG has bound itself to, and its heterodimerization or homodimerization partners, a downstream signaling cascade is activated via PI3K-AKT-mTOR pathway, RAS-RAF-MAPK, JAK-STAT, and PLCγ-PKC pathways (Fig. 1C) [21].

**Table 1:** NRG 1- 4 selected functions [1, 4, 8–20]

**NRG type**	**Normal function**	**Disease implications: cancer**	**Disease implications: neurologic/psychiatric and other disorders**	**References**
**NRG1**	-Influences normal neuronal function such as the development and maintenance of neurons and glia in the nervous system -Expressed in brain, heart, liver, kidneys, spinal cord, ovaries, and skin -Promotes myelination in glia-neuron interactions -Focused in the axonal areas of the neuron -Impacts enteric nervous system development -Influences expression of acetylcholine receptors in synaptic vesicles during neuromuscular junction formation -Affects growth of skeletal muscle cells -Impacts lobuloalveolar budding/milk production in the breast, differentiation of breast cancer cells, myocardium development	-Fusions related to malignancies includes NRG1-CD74, NRG1-SDC4, NRG1-CDH1 (refer to Fig. 1B** and **Tables 2 and 3) -NRG1 fusions enhance the function of NRG1	-Alterations in NRG1/ERBB4 signaling possibly related to amyotrophic lateral sclerosis (e.g., *ERBB4* I712M), schizophrenia and cognitive disorders such as Alzheimer’s disease -May be involved in Parkinson’s disease -Linked to cardiac development and disease -ERBB4/HER4 mutations in neurologic disease including amyotrophic lateral sclerosis are loss of function	[1, 4, 8–11, 20]
**NRG2** Also referred to as neural-and-thymus derived activator for ERBB kinases (NTAK)	-Expressed in the brain (mainly in the olfactory bulb, cerebellum, and hippocampus) and thymus -NRG-2, secreted from astrocytes, bound to ErbB3 on neurons and promoted neuronal survival -Regulates voltage gates ion channels and neurotransmitter receptors of neighboring neurons (i.e., astrocyte functions)	-CD74-NRG2alpha fusion in lung cancer has been described	-May be involved in schizophrenia	[1, 12–15, 20]
**NRG3**	-Neuronal development, proliferation, differentiation, and plasticity -NRG-3 has a key function in promoting early mammary morphogenesis	-NRG-3 is involved in breast cancer	-May be involved in Alzheimer’s disease and schizophrenia *-NRG3* mutations, especially those that result in overexpression, may cause symptoms synonymous to those seen in attention deficit hyperactive disorder, as well as broader cognition disorders.	[1, 16, 17, 20]
**NRG4**	-An adipocytokine in which proper expression levels can subvert non-alcoholic fatty liver disease -Aids brown adipose tissue in maintaining normal lipid levels within the liver. -Regulates glucose and lipid metabolism.		-Lowered expression may contribute to obesity-related disorders in a pleiotropic manner -NRG-4 expression was decreased in human inflammatory bowel disease samples and mouse models of colitis, suggesting that activation of ErbB4 is altered -May be involved in schizophrenia	[1, 18–20]

Recently, molecular alterations in ERBB4/HER4 receptor (loss-of-function) or in NRG1 have been linked to several neurological diseases such as amyotrophic lateral sclerosis (ALS) and schizophrenia [8, 9, 22]. Indeed, the *ERBB4/HER4* gene is designated *ALS19* in the neurologic literature [11]. Furthermore, *NRG1* genomic abnormalities (especially fusions, which enhance the activity of NRG1) have been found in advanced cancers. These discoveries could be therapeutically important for both cancer and non-cancer conditions (Fig. 1B) [23–25].

Herein we discuss the diverse role of NRG1 in various disease types, as well as possible implications for precision targeted therapeutics in both neurologic disease and cancer, based on cross-fertilization of knowledge from each field to the other [22, 26]. Additionally, we present a case of a woman with pancreatic cancer harboring a *VTCN1-NRG1* fusion whose tumor had progressed after multiple treatments but was responsive to pertuzumab and trastuzumab (anti-HER2 targeted antibodies).

## Main text

### Function of NRG

Under normal conditions, NRG1 has several important functions based on the specific isoform of the protein, with each NRG1 isoform contributing to the frictionless function of a complex neuronal network (Table 1). NRG1 types 1 and 3 are designed to maintain normal neuronal growth, especially during development [27]. These functions include processes such as the development of glial cells, synaptic plasticity, and synaptic transmissions [28]. An extension of this neuronal network includes the enteric nervous system that lines the gastrointestinal wall, and within the enteric nervous system are NRG1-positive neurons [29]. NRG1 is also expressed in the heart, liver, kidneys, spinal cord, ovaries, and skin, and multiple fusions have been found in cancer [25, 26]; it has also been linked to cardiac development and disease [3]. Taken together, NRG1–ERBB/HER signaling is critically important for neuronal progenitor proliferation, survival, maturation, and synapse formation, but is also multifunctional, and particularly important in cancer.

NRG2 expression remains quite localized within the brain and thymus regions where it promotes astrocyte survival and dendrite outgrowth [13, 14]. However, a CD74-NRG2 alpha fusion has been described in lung cancer [12]. Although further research is needed for NRG3, it seems to mirror the functionality of NRG1 by aiding neuronal development, differentiation, and plasticity [16, 17]. NRG3 also has a key function promoting normal breast development and may be involved in breast cancer [30, 31]. Lastly, NRG4 undertakes metabolic functionalities by maintaining normal lipid levels in the liver as an adipocytokine [19, 32]. NRG4 levels are also modified in inflammatory bowel disease models [33, 34].

### NRG1 and bidirectional signaling

NRG1 is a ligand for ERBB3/HER3 and ERBB4/HER4 and, hence, also influences EGFR/ERBB1/HER1 and ERBB2/HER2 receptor signaling via heterodimerization with ERBB3/HER3 and ERBB4/HER4 (Fig. 1A). Importantly, NRG1 signaling is bidirectional and quite complex (Fig. 1C–E). In traditional forward signaling, NRG1 stimulates the PI3K–Akt–S6K and the Raf–MEK–ERK pathways (Fig. 1C); in non-canonical forward signaling, ERBB4/HER4 undergoes cleavage resulting in release of an intracellular domain that can journey to the nucleus and control gene expression (Fig. 1D) [35, 36]. In the other direction — backward signaling — ERBB4/HER4 or its diffusible extracellular domain can act as a ligand for pro-NRG1 (Fig. 1E). To further complicate matters, the intracellular domain of pro-NRG1 also regulates transcription (Fig. 1E) [36].

### NRG-ERBB/HER pathway in ALS and other neurologic diseases: clinical and therapeutic implications

Mutations (loss of function) and other alterations of the ERBB4/HER4 receptor (also known as ALS19) have been linked to several neurological and psychiatric disorders, including ALS and schizophrenia (Table 1) [1, 4, 8–20, 37, 38]. Increasing recent evidence suggests that frontotemporal dementia and ALS also share some clinical, pathological, and molecular features as part of a common neurodegenerative spectrum disorder [9, 39].

Researchers have posited that loss-of-function mutated ERBB4/HER4 (ALS19) receptors do not properly auto-phosphorylate even in the presence of the NRG1 ligand [9]. Additionally, alteration of the NRG1-ERBB4/HER4/ALS19 pathway detrimentally affects motor neurons within the spinal cord in ALS [11]. While studies conducted by Takahashi et al. have attributed this pathogenesis model to familial ALS, which can carry germline mutations in *ERBB4/HER4/ ALS19* [37], the research group further applied this chain of events to sporadic ALS, which accounts for over 90% of ALS patients [11]. They found attenuated expression of ERBB4/HER4/ALS19 receptors, as assessed by immunohistochemistry, in the spinal cord of patients with sporadic ALS [11]. Decreased activity levels of ERBB4/HER4/ALS19 seem to be present in frontotemporal dementia as well, wherein Sun et al. showed minimal signaling when *ERBB4/HER4/ALS19* was mutated [9].

There is also growing evidence that aberrant NRG1 expression itself may be implicated in the pathogenesis of ALS [40]. The transgenic superoxide dismutase 1 (mSOD1) ALS mouse model, which partially recapitulates the phenotype of human ALS, has shown increased type I (secreted) NRG1 expression that could contribute to disease progression as it was associated with glial cell activation (though type III (membrane-bound) NRG1 expression was reduced in parallel with motor neuron loss) [40]. Similarly, plasma NRG1 levels (which correlate with cerebrospinal fluid levels) were found to be higher in patients with Alzheimer’s dementia as compared to neurologic controls (*p* < 0.001), further implicating the role of NRG1 in the pathogenesis of neurodegenerative diseases [41].

NRG1 may also participate in the pathogenesis of schizophrenia [8]; a marked increase in NRG1 signaling can be seen in the prefrontal cortex in schizophrenia and, moreover, NRG1 stimulation suppresses N-methyl-D-aspartate (NMDA) receptors (a family of L-glutamate receptors that play an important role in learning and memory) in the human prefrontal cortex in schizophrenia and in schizophrenic brain models [42].

The reason for NRG1 upregulation in ALS is unclear; we postulate that upregulation of the NRG1 ligand may occur as a feedback loop in response to attenuated ERBB4/HER4/ALS19 signaling due to loss-of-function mutation or dampened expression for other reasons and may in turn upregulate other ERBB receptors [9, 11]. Importantly, disease progression may be slowed in the mSOD1 mouse model of ALS by blocking neuregulin-induced microglial activation [43]. Of interest in this regard, in mSOD1 mice and in ALS patients, spinal cord microglial cells express the activated form of ERBB2 receptor and there are enhanced levels of NRG1 in microglial cells [40]. Conversely, the interactions may be more nuanced. For instance, other studies have shown decreased levels of NRG1 type III (membrane-bound form) and increased levels of NRG1 type I (secreted form) in the cerebrospinal fluid of patients with ALS, and the effects are mirrored in mSOD1 knockout mice [40, 44]. Further, reintroducing the NRG1 type III gene via a viral-vector restores neuromuscular function and improves survival in these knockout mice [45, 46].

Mutations of both *NRG2* and *NRG3* can also induce neuropathology. Though further studies are required, *NRG2* mutations seem to be related to mood dysfunction due to bipolar disorders and/or depression [47]. *NRG3* mutations, especially those that result in overexpression, are thought to cause symptoms synonymous to those seen in attention deficit hyperactive disorder (ADHD), as well as broader cognition disorders [16]. Mutations in either *NRG2* or *NRG3* may also correlate with schizophrenia; however, the latter is contingent on the maturity of the medial prefrontal cortex [15, 17]. Lastly, although mutations of *NRG4* may occur, they present as metabolic disorders rather than neurologic disorders [18, 19].

There are no currently FDA-approved therapies and no clinical trials that we could find for patients with neurologic or psychiatric disorders that address NRG1 or ERBB4/HER4/ALS19 alterations/perturbations. However, masitinib, a multikinase inhibitor (with activity against Kit, Lyn, PDGFR/Abl/Fms/Src, and FGFR3, some of which may signal downstream of the ERBB/HER system [48]) has shown activity in an mSOD1-mutant rodent model of ALS [49] and has demonstrated potential efficacy in a phase 2/3 clinical trial in ALS (NCT02588677) [50], a trial in primary progressive multiple sclerosis or nonactive secondary progressive multiple sclerosis [51], and Alzheimer’s disease [52, 53]. Importantly, a randomized, placebo-controlled phase 2/3 study of masitinib demonstrated that orally administered masitinib slowed rate of functional decline, with acceptable safety, in ALS patients and prolonged survival by over two years as compared with placebo, provided that treatment starts prior to severe impairment of functionality [50].

### NRG1 in malignancy

*NRG1* fusions can be found in diverse cancer types (Table 2), albeit at a low rate — ~0.15–0.5% across cancers (Table 2). Jonna et al. [54] reported that, among 21,858 patients with a variety of tumor types, although ultra-rare, *NRG1* fusions were detected in malignancies including sarcoma, non-small cell lung cancer, gallbladder, pancreatic, renal, ovarian, breast, bladder, and colorectal cancers, with several fusion partners observed (Table 2) [23, 29, 54–60]. These genomic fusions are a result of chromosomal inversions, insertions and deletions, or translocations [28, 61]. The hybrid gene is then able to bind to specific receptor types and initiate downstream cascades that often lead to deregulated activity. Various combinations of the NRG protein and receptor type lead to unique cellular pathway signaling. When gene fusions are involved, this can account for the aggressiveness of the cancer type as well as resistance to targeted therapeutics. An example is the gene fusion of *CD74*-*NRG1* in invasive mucinous adenocarcinoma of the lung; this specific fusion allows for a stronger affinity for receptor binding relative to other isoforms [61]. Pathways downstream of this fusion all contribute to deregulated activity within the cell.

**Table 2:** Examples of NRG1 fusions, their frequency, and partners, in various cancers (see also Fig. 2)

**Cancer type**	**Frequency of NRG1 fusions in designated cancer**	**Fusion partner(s) with NRG1**	**References**
All cancers	0.15–0.5%	Multiple—see below	[54]
Non-small cell lung cancer	0.3–0.8%	CD74 SDC4 SLC3A2 TNC MDK ATP1B1 DIP2B RBPMS MRPL13 ROCK1 DPYSL2 PARP8	[54–56]
Gallbladder	0.5%	NOTCH2 ATP1B1	[54]
Pancreatic ductal adenocarcinoma	0.5–1.2%	ATP1B1 CDH1 VTCN1	[54–56]
Renal cell carcinoma	0.5%	RBPMS	[54, 55]
Ovarian	0.4%	SETD4 TSHZ2 ZMYM2	[54, 55]
Breast	0.2–0.5%	ADAM9 COX10-AS1	[54–56]
Sarcoma	0.2%	WHSC1L1	[54, 55]
Bladder	0.1%	GDF15	[54, 55]
Colorectal	0.1%	POMK	[54, 55]
**NRG1 fusion partners and their relative frequency across cancers**			
**Fusion partner with NRG1**	**Frequency of fusion partner**		**References**
CD74	29–31%		[29, 54, 57, 58]
ATP1B1	10%		[23, 54, 59]
SDC4	7–11%		[54, 57]
RBPMS	2–5%		[54, 57, 59]
ADAM9	2%		[54, 59, 60]
CDH1	2%		[54, 60]
COX10-AS1	2%		[54, 60]
GDF15	2%		[54]
NOTCH2	2%		[54, 60]
POMK	2%		[54, 60]
SETD4	2%		[54, 60]
SLC3A2	2%		[29, 54, 59]
TSHZ2	2%		[54, 60]
VTCN1	2%		[54, 60]
WHSC1L1	2%		[54, 60]
ZMYM2	2%		[54, 60]
ROCK1	1–2%		[54, 59, 60]
MDK	1–2%		[54, 60]
MRPL13	1–2%		[54, 59, 60]
TNC	1–2%		[54, 59, 60]
DIP2B	1–2%		[54, 60]
PARP8	1–2%		[54, 59, 60]
DPYSL2	1–2%		[54, 59, 60]

### NRG1 and cancer therapeutics

*NRG1* fusion-bearing cancers may be therapeutically important. Fusions of several different gene types (e.g., *BCR-ABL*, *NTRK*, and *RET* fusions) are known drivers of cancer, and several successful therapies have been developed to target them [62–69]. Moreover, a recent study suggested that not targeting a fusion, if present, is associated with poorer clinical outcome even when genomic co-alterations are targeted [62].

NRG1 fusions are found in a variety of forms and cancers (Table 2) [23, 29, 54–61, 70–73]. For example, *NRG1* fusions with *CD74* are predominantly seen in non-small cell lung cancers (NSCLCs) [54, 55, 58, 59, 73], the *NOTCH2*-*NRG1* fusion in gallbladders cancers [54], and the *CDH1*-*NRG1* fusion in pancreatic ductal adenocarcinoma [26, 54, 55, 71, 72] (Table 2). NRG1 fusions have also been characterized in renal cell carcinoma, ovarian, breast, and some sarcomas [23, 54, 56, 61, 70]. Importantly, roughly 90% of patients with pancreatic ductal adenocarcinomas have a *KRAS* mutation but, for those patients without a *KRAS* mutation, an *NRG1* fusion can sometimes be found [26, 71, 72]. This is clinically significant since, although rare, NRG1 fusions can be targeted with HER-tyrosine kinase inhibitors such as afatinib (pan-HER inhibitor), trastuzumab (anti-HER2 antibody) or pertuzumab (antibody to the extracellular domain II of HER2 that attenuates ligand-dependent HER2–HER3 dimerization) (Fig. 1C) [26, 72]. Jones et al. reported two patients with *NRG1* fusion, one of which had lung adenocarcinoma and the other had cholangiocarcinoma, treated with afatinib who had durable responses [24]. Other investigators have suggested that irreversible pan-ERBB/HER inhibitors such as neratinib or lapatinib may also block impact of NRG1 [22, 74, 75]. Notably, another study [23] argued that anti-HER3 targeted therapy might be effective for NRG1 fusion tumors since NRG1 binds ERBB3/HER3–ERBB2/HER2 heterodimers and activates downstream signaling; they provided evidence of a durable response in an *NRG1*-rearranged invasive mucinous adenocarcinoma of the lung treated with the anti-ERBB3 monoclonal antibody (GSK2849330) [23]. Although *in vitro* data supported the use of either ERBB3 or ERBB2 inhibition, they saw more profound antitumor activity and downstream signaling inhibition with anti-ERBB3/HER3 versus anti-ERBB2HER2 therapy in an *NRG1*-rearranged patient-derived xenograft model [23]. Thus, cancers that harbor *NRG1* fusions may be treated with specific ERBB2/HER2 or ERBB3/HER3 or pan ERBB/HER pathway inhibitors.

Other drugs targeting the consequences of NRG1 fusions are currently under development. The HER2-HER3 bispecific antibody zenocutuzumab has received FDA fast track designation; it docks on ERBB2/HER2, then binds to, and blocks the NRG1 fusion-ERBB3/HER3 interaction and ERBB3/HER3 heterodimerization with ERBB2/HER2. The response rate was 34% and median duration of response of 9.1 months across multiple NRG1 fusion bearing solid tumors (e.g., NSCLC, pancreas cancer, breast cancer, cholangiocarcinoma) [76]. The anti-HER3 antibody seribantumab is also under development and was tested in a small multicenter phase 2 study, with most patients having NRG1 fusion NSCLC; the response rate was 30% [77].

Taken together, several drugs that target ERBB2/HER2 and/or ERBB3/HER3, including small molecule inhibitors and antibodies, have shown evidence of pan-cancer activity in NRG1 fusion bearing malignancies. Responses have been observed in multiple tumor types including, but not limited to lung, pancreatic, cholangiocarcinoma, and ovarian cancer and with a variety of fusion partners for NRG1 (Table 3) [61, 77–82].

**Table 3:** Examples of NRG1 targeting molecules and clinical trials and outcomes

**Molecule name**	**Type of molecule and mechanism**	**Clinical trial description**	**Outcomes**	**Comments**	**References/NCT number**
Zenocutuzumab	IgG1 bispecific antibody with enhanced ADCC activity targeting HER2 and HER3 receptors	Phase 1/2, open-label, multi-center, multi-national, dose escalation, single agent study to assess the safety, tolerability, PK, PD, immunogenicity, and anti-tumor activity of zenocutuzumab (MCLA-128) in patients with solid tumors harboring an NRG1 fusion (eNRGy)	ORR 34% and median duration of response of 9.1 months across multiple NRG1 fusion-bearing solid tumors (e.g., NSCLC, pancreas cancer, breast cancer, cholangiocarcinoma) Study ongoing	Patients with NRG1 fusions	[76]/NCT02912949
Seribantumab	Fully human anti-HER3 IgG2 monoclonal antibody	Phase 2, open-label, international, multi-center, study in adult patients with recurrent, locally-advanced or metastatic solid tumors, which harbor the NRG1 gene fusion (CRESTONE)	ORR was 30% (only 10 patients, with most having NSCLC)	Patients with NRG1 fusions	[77]/NCT04383210
Zenocutuzumab	Bispecific antibody with activity targeting HER2 and HER3 receptors	Phase 1/2 in advanced solid tumors	70% CBR in 10 patients with HER2+ metastatic breast cancer	NRG1 fusion not required for eligibility	[78]
GSK2849330	Anti-HER3 monoclonal antibody	Phase 1, first-in-human, open-label study assessed the safety, PK, PD, and preliminary activity of GSK2849330 in patients with HER3-expressing advanced solid tumors	Of 29 patients, 1 confirmed PR, for 19 months in a patient with CD74-NRG1-rearranged NSCLC	NRG1 fusion not required for eligibility	[79]
Tarloxotinib	Potent, covalent pan-HER tyrosine kinase inhibitor	Phase 2, open-label, single-treatment arm clinical trial in adult patients with NSCLC whose tumors test positive for EGFR exon 20 insertions (cohort A), HER2 mutations (cohort B), and NRG1 and HER fusions (cohort C)	No results for cohort C	Patients with a variety of alterations including NRG1 fusions	[81]
Afatinib	Irreversible pan-ERBB tyrosine kinase inhibitor	Novel, prospective real-world outcomes study based on single-patient protocol data in patients with advanced/metastatic solid tumors harboring NRG1 gene fusions	Study ongoing	Patients with NRG1 fusions	[80]
Zenocutuzumab	Bispecific antibody targeting HER2 and HER3 receptors	Phase 2, open-label, 2-arm, multicenter, international study designed to evaluate the efficacy of zenocutuzumab alone or in combination in patients with the following diagnoses: Group A: NRG1 fusion positive NSCLC Group B: mCRPC	Study ongoing	Patients with NRG1 fusion bearing NSCLC or with mCRPC (no NRG1 fusion required for the latter)	NCT05588609

*Abbreviations*: *ADCC*, antibody-dependent cell-mediated cytotoxicity, CBR was defined as CR + PR + SD ≥12 weeks, *CR*, complete response, *DCR*, disease control rate, *PD*, pharmacodynamics, *PK*, pharmacokinetics, *mCRPC*, metastatic castrate-resistant prostate cancer, *NSCLC*, non-small cell lung cancer, *ORR*, objective response rate, *POD*, progression of disease, *PR*, partial remission, *PSA50*, prostate-specific antigen level ≥ 50% response, *SD*, stable disease

## Case study: Patient with pancreatic cancer and *VTCN1-NRG1 *fusion (*KRAS* wild type) treated with trastuzumab and pertuzumab

**Fig. 2:**
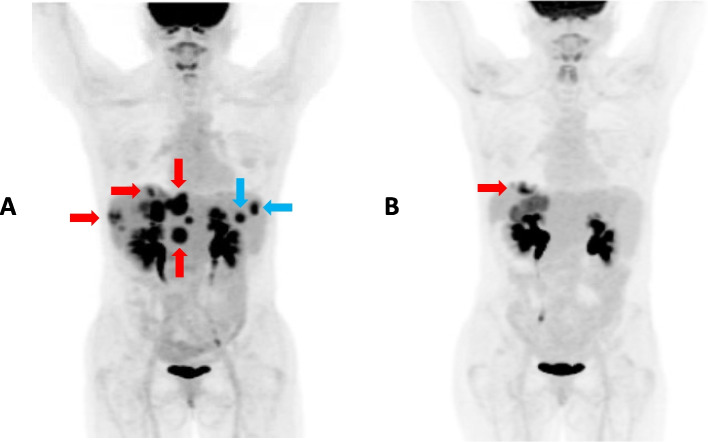
Imaging before panel **A** receiving anti-HER2 directed therapy and 6 months after panel **B** receiving trastuzumab, pertuzumab, and gemcitabine. Patient is a 47-year-old woman with NRG1 fusion (*VTCN1/NRG1)*, KRAS wild-type pancreatic cancer (whose disease had previously progressed on gemcitabine-based therapy). Panel **A** represents a scout film from PET imaging that shows innumerable hepatic lesions (red arrows), splenic metastases (blue arrows), normal tracer in the kidneys, brain, and urinary bladder before receiving anti-HER2 therapy. Panel **B** shows decreased in the number of liver lesions (red arrow), diminished splenic metastases, and redemonstrates normal tracer in the kidneys, brain, and urinary bladder, 6 months after receiving trastuzumab, pertuzumab, and gemcitabine

A 47-year-old woman with metastatic pancreatic cancer, who had progressed on multiple prior therapies including, but not limited to folinic acid, fluorouracil, irinotecan, and oxaliplatin, gemcitabine plus nab-paclitaxel, and a pembrolizumab-based treatment, had next-generation sequencing, which showed microsatellite-stable (MSS), tumor mutation burden (TMB) of 7 mutations/megabase, *CREBBP* exon 16 p.E1058fs, *PBRM1* exon 12 p.Y417fs, and a *VTCN1-NRG1* fusion (Caris Life Sciences and Ashion/Exact Sciences). She was started on trastuzumab (ERBB2/HER2 targeting antibody), pertuzumab (antibody that inhibits ligand-dependent ERBB2/HER2–ERBB3/HER3 dimerization), and gemcitabine. Trastuzumab and pertuzumab have shown synergy in breast cancer [82]. Her scans showed marked reduction in tumor size (Fig. 2) at 6 months. She tolerated the therapy well.

## Conclusions

### NRG1 and ERBB4 (ALS19) at the intersection between neurodegenerative disease and cancer

Neuregulins are a family of EGF-like signaling molecules that are implicated in the development and repair of diverse body elements including those of the nervous system, skeletal muscle, heart, breast, and other organs [1–3]. The neuregulin family includes NRG1 (types I–VI), NRG2, NRG3, and NRG4. The *NRG1* gene can translate to six different NRG1 protein types and over 30 different isoforms that act as extracellular EGF-like ligands for ERB3/HER3 and ERRB4/HER41. The numerous isoforms enable NRG1 to impact protean biologic functions, such as growth and differentiation of glial, neuronal, and Schwann cells, as well as skeletal muscle and mammary cells, and the myocardium [4].

Recently, molecular alterations in ERBB4/HER4/ALS19 receptors (loss of function) have been linked to several neurological diseases such as ALS and schizophrenia [8, 9, 11, 22, 37]. ALS is a devastating neurodegenerative disorder affecting primarily the motor system; there is loss of corticospinal neurons in the motor cortex, as well as in motor neurons in the anterior horn of the spinal cord, giving rise to progressive muscle weakness and wasting, with survival limited to 2 to 5 years [83].

Since some patients with ALS appear to have dampened ERBB4/ALS19 function in tissues of the central nervous system [8, 9, 22], either due to germline mutations or via other mechanisms [11, 37], it seems unexpected that even potent pan-ERBB/HER kinase inhibitors such as neratinib and dacomitinib that are used in the clinic to treat cancer do not have significant neurologic side effects, even though these pan-ERBB2/HER inhibitors attenuate ERBB4/HER4/ALS19 function, and can be continued for months or years for cancer treatment [84, 85]. This observation suggests that it is plausible that ERBB4/HER4/ALS19 dampened activity in of itself may not be enough to cause neurodegeneration. Of interest in this respect, there is accumulating evidence that aberrant NRG1 expression (in addition to the loss-of-function *ERBB4/HER4/ALS19* mutations) may be implicated in the pathogenesis of ALS [40]; murine models have shown increased type I (secreted) NRG1 expression that could contribute to disease progression via glial cell over-stimulation in ALS. The reason for NRG1 upregulation is unclear; we postulate that upregulation of the NRG1 ligand may occur as a feedback loop in response to reduced ERBB4/HER4/ALS19 signaling caused by loss-of-function *ERBB4/HER4/ALS19* mutations or dampened ERBB4/HER4/ALS19 expression that occurs for other reasons in ALS [9, 11, 37]. Perhaps the heightened NRG1 expression in the presence of ERBB4/HER4 loss overstimulates ERBB1/HER1, ERBB2/HER2 or ERBB3/HER3 in neuronal tissue, leading to damage. Indeed, in mSOD1 ALS model mice and in ALS patients, spinal cord microglial cells express the activated form of ERBB2/HER2 receptor and there were enhanced levels of NRG1 in microglial cells [40]. When patients with cancer are given pan-ERBB/HER inhibitors, ERBB4/HER4/ALS19 function is attenuated, but any feedback upregulation of NRG1 should it occur cannot overstimulate ERBB1/2/3 because the pan-ERBB/HER inhibitor diminishes the function of these other ERBBs. It is therefore conceivable that pan-ERBB/HER inhibition should be investigated for ALS, preclinically or clinically in a subset of patients who may have altered ERBB or NRG1 function. Notably, a randomized, placebo-controlled phase III study of masitinib (which targets multiple kinases, including some that may be downstream of the ERBB/HER receptors) demonstrated slowed rate of functional decline, with acceptable safety, in ALS patients, and prolonged survival by over two years as compared with placebo, provided that treatment started prior to severe impairment of functionality [50]. However, it should be kept in mind that neurodegenerative diseases are complex and heterogenous and other mechanisms such as messenger RNA translation defects might be operative in patients [86]. Indeed, there is an increasing appreciation that ALS is a heterogenous disorder; further biomarker analysis of ALS populations may yield subsets of patients whose disease may be susceptible to pan-ERBB/HER inhibition.

In the cancer realm, *NRG1* genomic abnormalities (especially fusions that result in enhanced function) have been found in advanced cancers, a discovery which could be therapeutically important (Fig. 1C, Tables 2 and 3) [23–25]. Multiple small molecule and antibody inhibitors that impact the ERBB3/ERBB4 axis upregulated in the face of *NRG1* fusions are under investigation. Although NRG1 fusions are rare in cancer, occurring in 0.15-0.5% of malignancies, they can be found in multiple types of cancer and, in addition, NRG1 may have numerous fusion partners (Table 2). Emerging preliminary data suggests that NRG1 fusion-bearing malignancies are susceptible to targeting by pan-ERBB/HER small molecule inhibitors (including afatinib) and by antibodies that impact ERBB3/ERRB4 and/or dimerization partners such as ERBB2/HER2 (e.g., zenocutuzumab and seribantumab and, as in our illustrative *VCTN1-NRG1* fusion/*KRAS* wild-type pancreatic cancer patient, with trastuzumab and pertuzumab) (Table 3, Fig. 2). Responses appear to occur in a tumor-agnostic fashion and have been described in *NRG1* fusion-bearing lung, cholangiocarcinoma, ovarian, and pancreatic cancers, suggesting that malignancies harboring *NRG1* fusions merit further investigation for another tissue-agnostic approval [67, 87].

In summary, disruption of the NRG1/ERBB4 (ALS19) axis offers therapeutic possibilities for both cancer and neurologic diseases such as ALS. In the cancer realm, suppression of this axis is being tested and shows promising results in patients whose tumors harbor *NRG1* fusions and similar alterations leading to aberrant activation/expression*.* In ALS, the interactions may be more complex, but current data suggest the possibility that dampened ERBB4 (ALS19) function could lead the upregulation of NRG1 and secondary overstimulation of other ERBB (HER) receptors; hence, clinical trials of pan-ERBB/HER inhibitors, currently in use and approved for cancer, merit investigation in ALS.

## Data Availability

All relevant data is presented within the publication.

## Abbreviations

ADCC: Antibody-dependent cell-mediated cytotoxicity
ALS: Amyotrophic lateral sclerosis
CBR: Clinical benefit rate
CD: Cytoplasmic domain
CR: Complete response
CRD: Cysteine-rich domain
DCR: Disease control rate
ECD: Extracellular domain
EGF: Epidermal growth factor
EGF-L: EGF-like repeat
HB-EGF: Heparin-binding EGF-like growth factor
Ig: Ig-like C2-type domain
LIMK: LIM kinase
mCRPC: Metastatic castrate-resistant prostate cancer
mSOD1: Transgenic superoxide dismutase 1
N-CoR: Nuclear receptor co-repressor
NMDA: N-methyl-D-aspartate
NRG1: Neuregulin-1
NSCLC: Non-small cell lung cancer
ORR: Objective response rate
PD: Progressive disease
PK: Pharmacokinetics
PR: Partial response
PSA50: Prostate-specific antigen level ≥ 50%
SD: Stable disease
TAB2: TGF-Beta Activated Kinase 1
TGF: Transforming growth factor
TM: Transmembrane
TMD: Transmembrane domain
WT: Wild type
